# More bullets for PISTOL: linear and cyclic siloxane reporter probes for quantitative ^1^H MR oximetry

**DOI:** 10.1038/s41598-020-57889-9

**Published:** 2020-01-29

**Authors:** Shubhangi Agarwal, Praveen K. Gulaka, Ujjawal Rastogi, Vikram D. Kodibagkar

**Affiliations:** 1 School of Biological and Health Systems Engineering, Arizona State University, Tempe, AZ 85295 USA; 2 Department of Radiology, University of Texas Southwestern Medical Center at Dallas, Dallas, TX 75390 USA

**Keywords:** Magnetic resonance imaging, Prognostic markers, Biomedical materials

## Abstract

Tissue oximetry can assist in diagnosis and prognosis of many diseases and enable personalized therapy. Previously, we reported the ability of hexamethyldisiloxane (HMDSO) for accurate measurements of tissue oxygen tension (pO_2_) using Proton Imaging of Siloxanes to map Tissue Oxygenation Levels (PISTOL) magnetic resonance imaging. Here we report the feasibility of several commercially available linear and cyclic siloxanes (molecular weight 162–410 g/mol) as PISTOL-based oxygen reporters by characterizing their calibration constants. Further, field and temperature dependence of pO_2_ calibration curves of HMDSO, octamethyltrisiloxane (OMTSO) and polydimethylsiloxane (PDMSO) were also studied. The spin-lattice relaxation rate R_1_ of all siloxanes studied here exhibited a linear relationship with oxygenation (R_1_ = A′ + B′*pO_2_) at all temperatures and field strengths evaluated here. The sensitivity index η( = B′/A′) decreased with increasing molecular weight with values ranged from 4.7 × 10^−3^–11.6 × 10^−3^ torr^−1^ at 4.7 T. No substantial change in the anoxic relaxation rate and a slight decrease in pO_2_ sensitivity was observed at higher magnetic fields of 7 T and 9.4 T for HMDSO and OMTSO. Temperature dependence of calibration curves for HMDSO, OMTSO and PDMSO was small and simulated errors in pO_2_ measurement were 1–2 torr/°C. In summary, we have demonstrated the feasibility of various linear and cyclic siloxanes as pO_2_-reporters for PISTOL-based oximetry.

## Introduction

Adequate availability of oxygen is critical to the efficient functioning of many vital organs and tissues^1^. Changes in oxygenation are indicative of a disruption in homeostatic conditions which are prevalent in pathologies such as tumors^2^, wounds^3,4^, ischemic heart disease^5,6^ metabolic disorders^7–9^ and traumatic brain injury^10^. The oxygen requirement changes between cells, tissues and organs and thus each tissue type exhibits a distinct normal range of oxygenation. For example, the normal tissue oxygen level in the brain is ~34 torr (mmHg) while that in the muscle is ~29 torr^11^. The lack of adequate oxygen in cells and tissues is termed as hypoxia and could result from diminished blood flow, low blood oxygen saturation, elevated oxygen metabolism and increased cellular proliferation. Oxygen homeostasis and hypoxic stress are being recognized as important factors for development and physiology of cells and tissues. These factors also influence the pathophysiology of diseases as they regulate various intracellular signaling pathways for processes such as angiogenesis, cell proliferation and protein synthesis^12–18^. Malignant tumors are known to have regions with low oxygen tension known as hypoxia which is a major driving force behind tumor progression and resistance to therapies^19–21^. Hypoxia presents itself as an ideal target for the development of anti-cancer therapies due to the role that it plays in the progression of cancer^22^. Thus, measurement of oxygen is essential for monitoring the function of organs as well as for diagnosis, treatment planning and studying treatment response of pathologies. Consequently, there is an increased need for an oximetry technique that can facilitate repeated, non-invasive and accurate assessment of oxygen and can be translated to the clinic.

Many qualitative and quantitative oximetry techniques have been developed for oximetry such as polarographic needle electrode^23^, fiber optic probes^23^, Near Infrared (NIR) spectroscopy^24^, fluorescence^25^, immunohistochemical probes^26^, positron emission tomography (PET)^27^ and single photon emission computed tomography (SPECT)^28^. Polarographic needle electrode and fiber optic probe techniques are invasive, susceptible to pressure artifacts and cannot facilitate simultaneous measurement of multiple locations and repeated measurements, while immunohistochemical hypoxia probes (pimonidazole^29^, EF5^30^, HIF1α^26^) are limited to *ex-vivo* analysis. NIR spectroscopy is a non-invasive technique but can only detect the changes in vascular oxygen saturation and cannot distinguish between signals from oxyhemoglobin, deoxyhemoglobin, and cytochrome *c* oxidase^31^. PET and SPECT based techniques lack spatial resolution and cannot provide quantitative information regarding oxygenation. The current magnetic resonance imaging (MRI) based oximetry techniques can be further sub-divided into a) qualitative techniques: Blood Oxygen Level Dependent (BOLD)^32^, Tissue Oxygen Level Dependent (TOLD)^33^, oxygen-enhanced MRI^34^, hypoxia targeted MRI^35^ and b) quantitative oximetry techniques: Electron Paramagnetic Resonance (EPR^36^), ^19^F NMR of perfluorocarbon emulsions, Fluorocarbon Relaxometry using Echoplanar imaging for Dynamic Oxygen Mapping (FREDOM, ^19^F MRI of hexafluorobenzene)^37^ and Proton Imaging of Siloxanes for mapping Tissue Oxygenation Levels (PISTOL)^38,39^).

EPR and MR oximetry (^19^F and ^1^H) techniques are minimally invasive and provide quantitative oxygenation information via measuring the change in linewidth or spin lattice relaxation time, respectively, of an exogenously administered paramagnetic spin probe as it interacts with the molecular oxygen. They allow for non-invasive and repeated measurement of oxygenation at multiple locations. Some of the EPR probes are lithium phthalocyanine (LiPc), lithium naphthalocyanine (LiNc), Fusinite, Gloxy, India Ink and triarylmethyl (TAM), of which India Ink is approved for clinical use^36,40^. The ^19^F MR oximetry uses exogenous perfluorocarbon reporters such as perfluoro-15-crown-5-ether (15C5^41^) or hexafluorobenzene^37,42,43^ as oxygen reporters and uses the linear relationship of flurocarbon spin-lattice relaxation rate R_1_ with oxygenation. Exploiting the same concept, our group has previously shown the feasibility of accurate and repeated measurements of oxygen using hexamethyldisiloxane (HMDSO) in thigh muscle and tumor regions *in vivo* using ^1^H MR oximetry^38,39^. We have also shown the ability of siloxane based nanoemulsions for tissue oximetry^44^ as well as cellular oximetry^45,46^. Siloxanes can be synthesized in a variety of forms (linear or cyclic, increasing chain length, with or without functional groups) and have been used in various applications such as biomedicine, cosmetics, and food processing^47^. While HMDSO has been shown to be a reliable pO_2_ reporter and has a large dynamic range and high pO_2_ sensitivity^38^, the values of spin lattice relaxation time T_1_ (= 1/R_1_) under hypoxic conditions can be as long as 11 s, leading to long measurement times. This raises the question whether any of the other siloxanes could be used as pO_2_ reporter molecules and how chain length and structure (linear versus cyclic) influence the pO_2_ sensitivity and dynamic range of T_1_ exhibited under different oxygenation conditions. In this study, we have characterized the calibration curves of various low molecular weight linear and cyclic siloxanes and assessed their utility as pO_2_ sensing reporter molecules for use with ^1^H MR oximetry. The siloxanes investigated here are: linear siloxanes HMDSO, octamethyltrisiloxane (OMTSO), decamethyltetrasiloxane (DMTSO), dodecamethylpentasiloxane (DDMPSO), trimethylsiloxy-terminated polydimethylsiloxane (PDMSO, M.W. 410) and cyclic siloxanes octamethylcyclotetrasiloxane (OMCTSO) and decamethylcyclopentasiloxane (DMCPSO). These siloxanes are commercially available, inexpensive and have a single ^1^H resonance around 0.1 ppm^48^. Further, field and temperature dependence of the pO_2_ calibration curves of HMDSO, OMTSO and PDMSO were also studied.

## Theory

Quantitative MR oximetry exploits the Fermi contact interactions between paramagnetic oxygen and reporter molecules^49^ that leads to a O_2_ concentration (and hence pO_2_) dependent relaxation of the nuclear spins. Probes used for *in vivo* MR oximetry must preferably possess the following characteristics: high oxygen solubility, hydrophobicity (so that diffusion of aqueous ions is restricted), should have a single resonance so that there are no chemical shift artifacts in the MR images and minimal dependence of R_1_ on temperatures. Due to paramagnetic nature of molecular oxygen, it tends to shorten the nuclear relaxation times and relaxes the nuclear spins faster thereby increasing the spin-lattice (longitudinal) and spin-spin (transverse) relaxation rate R_1_ and R_2_ respectively of the reporter molecule. The principle is based on the linear dependence of pO_2_ on the spin-lattice relaxation rate of the probe.

If x is the molar fraction of oxygen the net spin lattice relaxation rate R_1_ is given by^49^

1 $${R}_{1}=(1-x)\ast {R}_{1d}+x\ast ({R}_{1d}+{R}_{1p})={R}_{1d}+x\ast {R}_{1p}$$

Where R_1d_ = diamagnetic or anoxic component of the relaxation rate and

R_1p_ = paramagnetic component of the relaxation rate due to the contribution of oxygen.

As per Henry’s law, the dissolved mole fraction *x* is directly related to the partial pressure of oxygen.

2 $$p{O}_{2}=k\ast x$$

k is a constant that determines the solubility of oxygen in the agent and is different for different agents. Thus, net relaxation rate becomes

3 $${R}_{1}=A^{\prime} +B^{\prime} \ast p{O}_{2}$$

where A′ = R_1d_ and B′ = R_1p_/k

Since longitudinal relaxation rate is a function of temperature we assume a linear dependence of constants A′ and B′ on temperature (for relevant physiological range) which empirically can be defined as

4 $$A^{\prime} =A+C\ast T$$

5 $$B^{\prime} =B+D\ast T$$

Substituting value of A′ and B′ in Eq. [3] results in a temperature-dependent model for net relaxation rate

6 $${R}_{1}=A+B\ast p{O}_{2}+C\ast T+D\ast T\ast p{O}_{2}$$

Inverting the above equation, quantitative pO_2_ levels can be determined more accurately and reliably if temperature is also monitored.

7 $$p{O}_{2}=\frac{{R}_{1}-A-C\ast T}{B+D\ast T}$$

Therefore, estimation of these parameters (A, B, C and D) is crucial for accurate pO_2_ measurement. Equation [7] also allows us to relate the errors in pO_2_ estimation at a particular oxygenation level and temperature to the potential errors or uncertainty in temperature measurement, particularly for *in vivo* applications. Also, for a given R_1_ measurement, the relative error in pO_2_ determination per 1 °C error in temperature estimation at a particular temperature T and oxygenation level pO_2_ can be derived as:

8 $$\frac{\Delta p{O}_{2}}{\Delta T}=\frac{|C+D\ast p{O}_{2}|}{|B+D\ast T|}$$

While comparing signals from same volumes of various siloxanes (as typically a fixed volume of reporter probe would be used *in vivo*), the relative theoretical signals, α^siloxane^, of each (compared to HMDSO) can be computed by accounting for the density differences and the mole fraction of protons in a mole of the siloxane.

9 $${\propto }^{siloxane}=\frac{{\rho }^{siloxane}\ast {N}_{H}^{siloxane}\ast M{W}^{HMDSO}}{{\rho }^{HMDSO}\ast {N}_{H}^{HMDSO}\ast M{W}^{siloxane}}$$

where, ${\rho }^{siloxane}$ is the density of the siloxane under consideration (0.764 g/ml for HMDSO at 25 °C), $\,{N}_{H}^{siloxane}$ is the number of H atoms per molecule (18 for HMDSO) and $M{W}^{siloxane}$ is the molecular weight of the siloxane (162 g/mol for HMDSO). The theoretical values of α^siloxane^ are listedin Table 1.

**Table 1:** Summary of calibration constants and α^siloxane^ of the various linear and cyclic siloxanes at 4.7 T (37 °C).

Siloxane	Molecular wt. (g/mol)	Intercept A′ (s^−1^)	Slope B′ (s*torr)^−1^	η (X10^−3^) = B′/A′	Relative Signal (α^siloxane^)
HMDSO*	162.4	0.1125 ± 1.38 × 10^−3^	0.0013 ± 2.09 × 10^−5^	11.6	1.00
OMTSO	236.5	0.1597 ± 7.50 × 10^−3^	0.0012 ± 8.38 × 10^−5^	11.4	0.98
DMTSO	310.7	0.1780 ± 3.30 × 10^−3^	0.0015 ± 3.60 × 10^−5^	8.4	0.97
DDMPSO	384.8	0.2062 ± 6.70 × 10^−3^	0.0017 ± 7.44 × 10^−5^	8.2	0.97
OMCTSO	296.6	0.2827 ± 3.50 × 10^−3^	0.0016 ± 3.87 × 10^−5^	5.6	0.91
DMCPSO	370.8	0.3169 ± 3.10 × 10^−3^	0.0015 ± 3.47 × 10^−5^	4.7	0.92

*HMDSO data from ref. ^38^ (Kodibagkar *et al*., *Magn Reson Med* **55**, 743-748) reproduced here for comparison.

## Results

### Calibration curves of linear and cyclic siloxanes at 4.7 T

**Figure 1:**
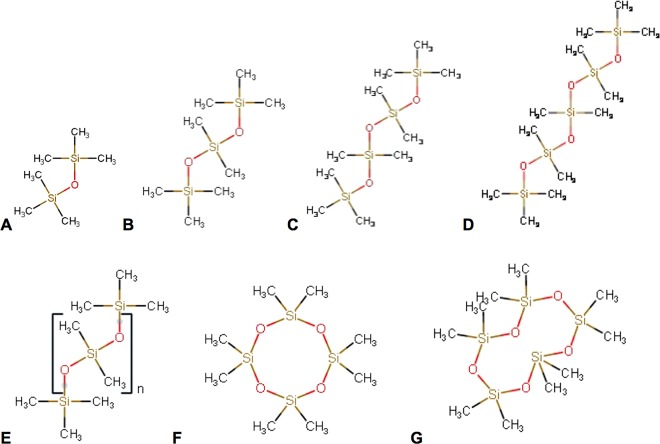
2D structures of the various linear and cyclic siloxanes characterized in this study. Linear siloxanes: (**A**) hexamethyldisiloxane (HMDSO), (**B**) octamethyltrisiloxane (OMTSO), (**C**) decamethyltetrasiloxane (DMTSO), (**D**) dodecamethylpentasiloxane (DDMPSO), (**E**) polydimethylsiloxane (PDMSO). Cyclic siloxanes: (**F**) octamethylcyclotetrasiloxane (OMCTSO) and (**G**) decamethylcyclopentasiloxane (DMCPSO).

**Figure 2:**
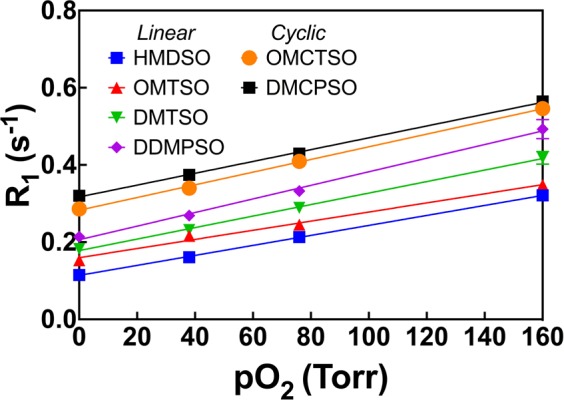
Dependence of spin lattice relaxation rate R_1_ on pO_2_ (at 37 °C and 4.7 T) for linear and cyclic siloxanes of different chain lengths. The cyclic compounds displayed higher longitudinal relaxation rates than the linear compounds at all oxygen concentrations. *Data from ref 38 (Kodibagkar *et al*., Magn Reson Med **55**, 743–748) reproduced here for comparison.

R_1_ of the linear and cyclic siloxanes (Fig. 1) OMTSO, DMTSO, DDMPSO, OMCTSO and DMCPSO were measured as a function of pO_2_ at 4.7 T and 37 °C and fit to the Eq. [3] to yield the calibration constants A′ and B′. At a fixed temperature (37 °C), the R_1_ of all the siloxanes showed a linear dependence on pO_2_ (R^2^ > 0.99) (Fig. 2). In the linear siloxanes, it was observed that with increasing molecular weight, the intercepts of the linear fits increased (ranging from 0.11–0.32 s^−1^) but the slopes were almost similar (ranging from 1.3 × 10^−3^ −1.7 × 10^−3^ s^−1^.torr^−1^). The cyclic compounds had higher R_1_ than the linear compounds at each oxygen concentration and showed a similar trend with increasing molecular weight as the linear siloxanes. The recovery curves for all siloxanes showed a monoexponential behavior (Supplementary Fig. S1). Table 1 lists the values of the calibration constants A′ and B′ for all the linear and cyclic siloxanes at 4.7 T and 37 °C along with the α^siloxane^ values. HMDSO calibration constants at 4.7 T were included from our previously published work^38^ for comparison.

### Temperature and field dependence of calibration of siloxanes

**Figure 3:**
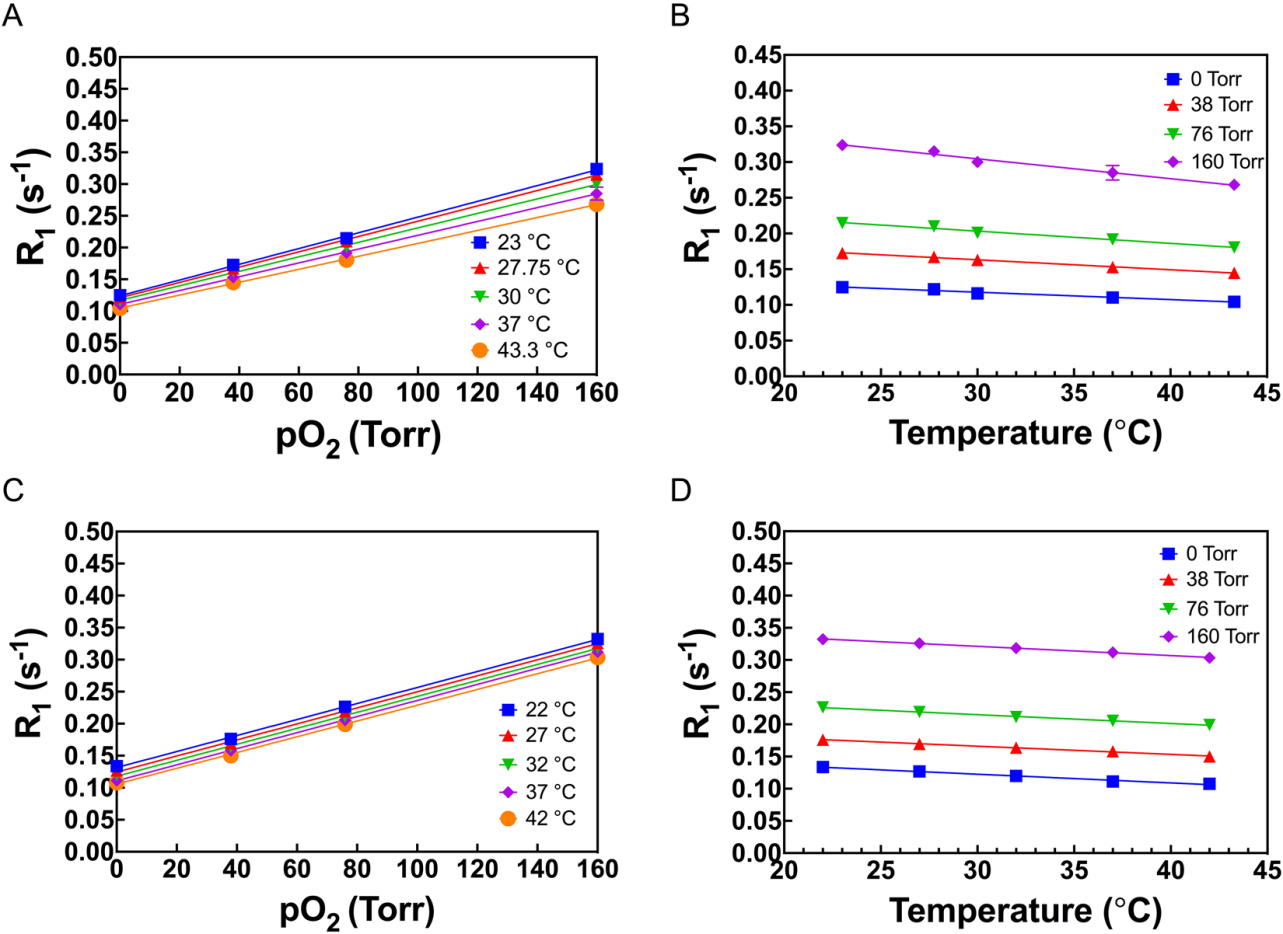
Dependence of spin lattice relaxation rate R_1_ of HMDSO at 7 T (**A**,**B**) and 9.4 T(**C,D**) on pO_2_ (**A**,**C**) and temperature (**B**,**D**).

The dependence of R_1_ of HMDSO on pO_2_ was determined at 7 T (Fig. 3A,B) and 9.4 T (Fig. 3C,D) as a function of temperature and was observed to be linear at both fields at all temperatures measured. Constants A′ and B′ were then plotted with respect to variations in temperature, in the physiologically relevant range of 17 °C to 48 °C, to yield characterization parameters A, B, C and D (Eqs. [4] and [5]) at 7 T (Supplementary Fig. S2A,B) and 9.4 T (Supplementary Fig. S2C,D). These are listed in Table 2. Relative pO_2_ error (Eq. [8]) was computed based on these constants and was found to be between 0.6–1 torr/°C in the physiologically relevant pO_2_ range 0–50 torr at 7 T (Supplementary Fig. S2E). In contrast, the relative pO_2_ error was ~ 1 torr/°C at 9.4 T showing very small variation between 0–50 torr (Supplementary Fig. S2E). E.g. for a pO_2_ of 5 torr, resulting error in pO_2_ determination per degree change in temperature at 37 °C was ~ 0.7 torr/°C at 7 T and ~ 1 torr/°C at 9.4 T.

**Table 2:** Summary of temperature dependence of calibration constants of siloxanes measured at different fields.

Siloxane	Field	A (s^−1^)	B (s*torr)^−1^	C (s*°C)^−1^	D (s*torr*°C)^−1^	Intercept A′ (s^−1^) 37 °C	Slope B′ (s*torr)^−1^ 37 °C	η (X10^−3^) = B′/A′
HMDSO	4.7 T*	0.1479 ± 2.8 × 10^−3^	0.0018 ± 5 × 10^−5^	−9.5 × 10^−4^ ± 8.1 × 10^−5^	−1.23×10^−5 ± ^1.3 × 10^−6^	0.1125 ± 1.38 × 10^−3^	0.0013 ± 2.09 × 10^−5^	11.6
HMDSO	7 T	0.1472 ± 1.34 × 10^−3^	0.0015 ± 2.4 × 10^−5^	−9.75 × 10^−4^ ± 3.9 × 10^−5^	−1.12 × 10^−5^ ± 6.9 × 10^−7^	0.1105 ± 1.05 × 10^−3^	0.00108 ± 1.16 × 10^−5^	9.77
HMDSO	9.4 T	0.1598 ± 8.78 × 10^−4^	0.0013 ± 1.75 × 10^−5^	−1.3 × 10^−3^ ± 2.8 × 10^−5^	−1.41 × 10^−6^ ± 5.67 × 10^−7^	0.1107 ± 5.04 × 10^−4^	0.00125 ± 5.57 × 10^−6^	11.3
OMTSO	4.7 T	0.2145 ± 1.28 × 10^−2^	0.002 ± 1.65 × 10^−4^	−1.34 × 10^−3 ± ^3.6 × 10^−4^	−2.84 × 10^−5^ ± 4.65 × 10^−6^	0.1597 ± 7.59 × 10^−3^	0.0012 ± 8.38 × 10^−5^	7.5
OMTSO	7 T	0.2030 ± 1.95 × 10^−3^	0.0017 ± 1.32 × 10^−5^	−1.49 × 10^−3^ ± 5.73 × 10^−5^	−1.17 × 10^−5^ ± 3.87 × 10^−7^	0.1484 ± 3.24 × 10^−3^	0.0013 ± 3.58 × 10^−5^	8.76
OMTSO	9.4 T	0.1990 ± 1.76 × 10^−3^	0.0016 ± 1.72 × 10^−5^	−1.37 × 10^−3^ ± 5.7 × 10^−5^	−8.37 × 10^−6^ ± 5.58 × 10^−7^	0.1477 ± 4.42 × 10^−3^	0.0013 ± 4.88 × 10^−^	8.8
PDMSO	7 T	0.2943 ± 2.53 × 10^−3^	0.0015 ± 2.61 × 10^−5^	−0.0023 ± 7.43 × 10^−5^	−7.9 × 10^−6^ ± 7.68 × 10^−7^	0.2085 ± 1.37 × 10^−3^	0.0012 ± 1.511 × 10^−5^	5.75

*HMDSO data from ref. ^38^ (Kodibagkar *et al*., *Magn Reson Med* **55**, 743–748) reproduced here for comparison.

**Figure 4:**
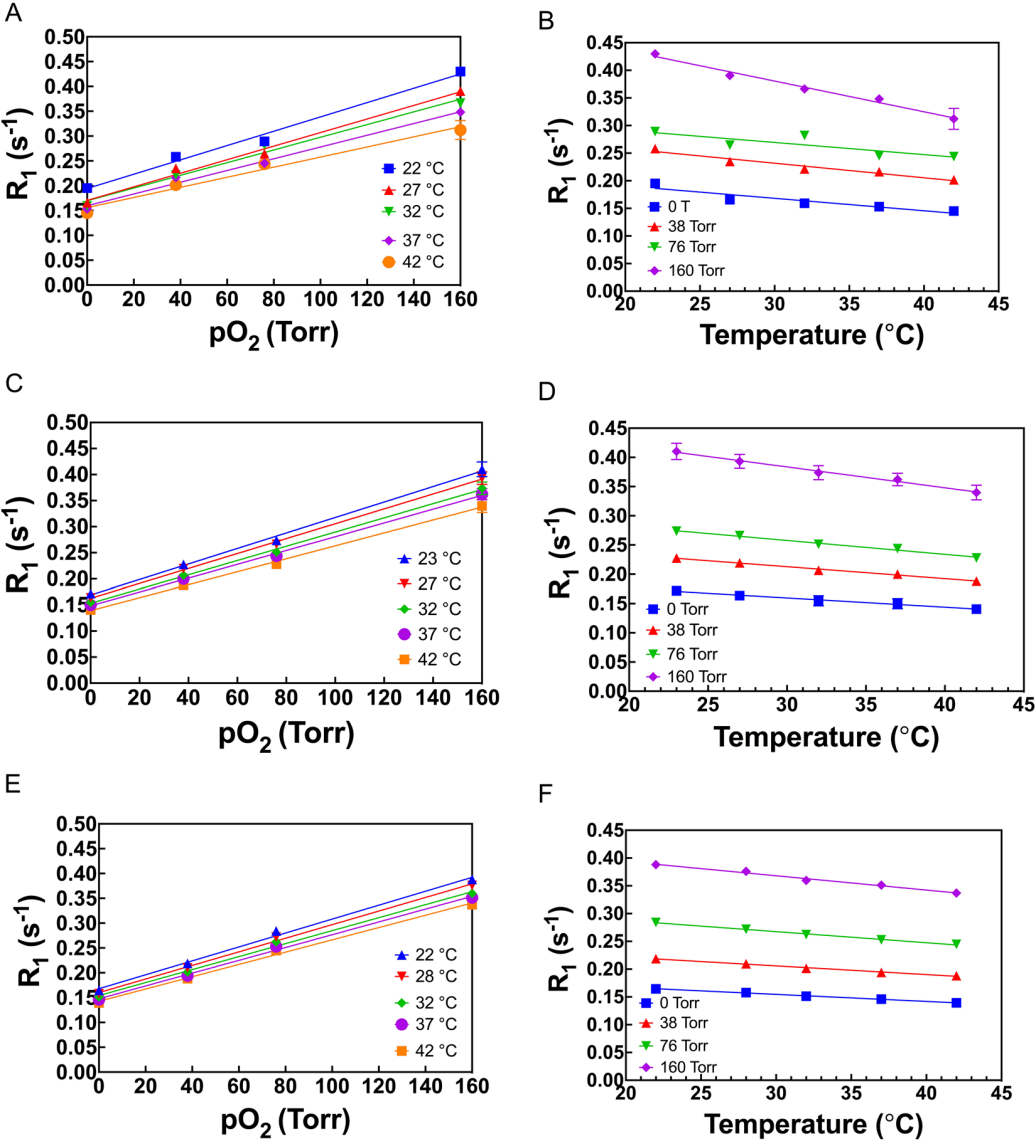
Dependence of spin lattice relaxation rate R_1_ of OMTSO at 4.7 T (**A**,**B**), 7 T (**C**,**D**) and 9.4 T (**E**,**F**) on pO_2_ (**A**,**C**,**E**) and temperature (**B**,**D**,**F**).

**Figure 5:**
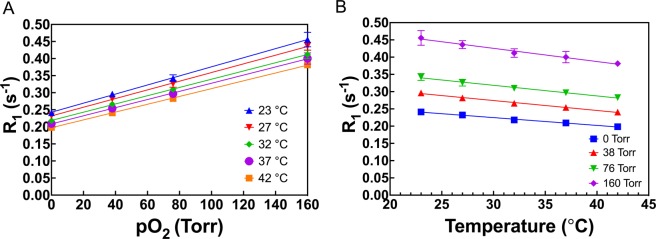
Dependence of spin lattice relaxation rate R_1_ of PDMSO on (**A**) pO_2_ and temperature at (**B**) 7 T.

**Figure 6:**
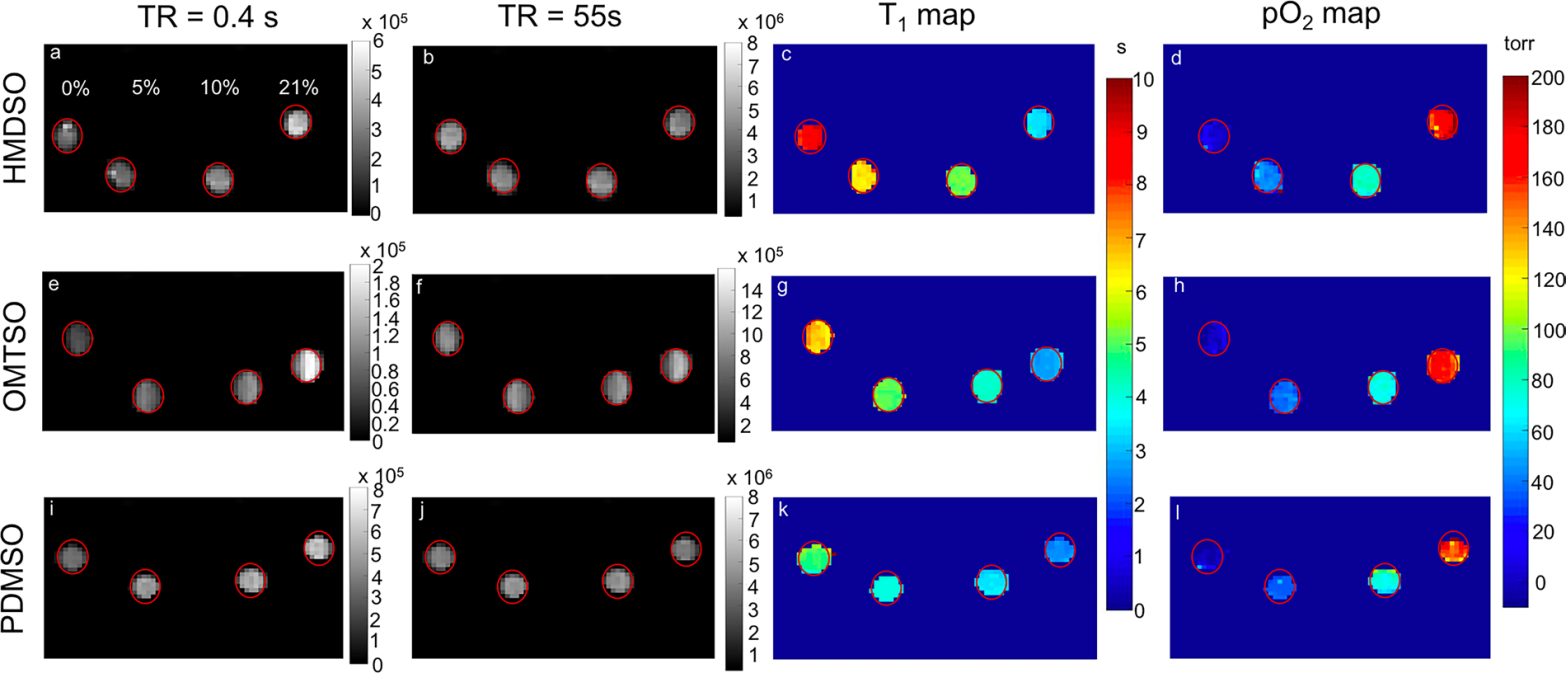
Representative MR images and T_1_ maps from the pO_2_ calibration along with predicted pO_2_ maps for HMDSO (**a**–**d**), OMTSO (**e**–**h**) and PDMSO (**i**–**l**) at 37 °C and 7 T. In each figure the tubes from left to right were bubbled with 0%, 5%, 10% and 21% O_2_ (balance N_2_), respectively. All the images were analyzed using MATLAB R2018b (MathWorks, https://www.mathworks.com/products/matlab.html).

The dependence of R_1_ of OMTSO on pO_2_ was determined at 4.7 T (Fig. 4A,B), 7 T (Fig. 4C,D) and 9.4 T (Fig. 4E,F) and was also observed to be linear at both fields and all temperatures studied. The temperature dependence of constants A′ and B′ was determined by a linear fit of the constants at different temperatures at 4.7 T, 7 T and 9.4 T (Supplementary Fig. S3A–F). Relative pO_2_ error for OMTSO ranged between 0.85–1.12 torr/°C in the physiologically relevant pO_2_ range at 9.4 T and was observed to be between 0.65–1.35 torr/°C at 4.7 T and ~ 0.87–1.2 torr/°C at 7 T (Supplementary Fig. S3G). E.g. relative pO_2_ error was calculated to be ~ 0.7 torr/°C, 0.9 torr/°C and 0.88 torr/°C at fields of 4.7 T, 7 T and 9.4 T, respectively, for a pO_2_ of 5 torr. The dependence of R_1_ of PDMSO on pO_2_ was determined at 7 T (Fig. 5) and was also found to be linear at all temperatures, as observed for HMDSO and OMTSO. Similarly, a linear fit of A′ and B′ at different temperatures yielded constants A, B, C and D for PDMSO at 7 T (Supplementary Fig. S4, Table 2). Based on these constants, the relative pO_2_ error was found to be between 1.5–1.8 torr/°C in the physiological pO_2_ range (Supplementary Fig. S4C). E.g. the relative error was calculated to be ~ 1.5 torr/°C for a pO_2_ of 5 torr at 7 T. With a view to compare the predicted pO_2_ values from the calibration constants with the actual pO_2_, we computed pO_2_ maps from the calibration data at 7 T and 37 °C (using the corresponding constants shown in Table 2) for HMDSO, OMTSO and PDMSO. These are shown in Fig. 6 and match the target pO_2_ (0, 5, 10 and 21% O_2_ corresponding to 0, 38, 76 and 160 torr respectively) used to bubble the siloxanes in the corresponding tubes.

## Discussion

HMDSO has been previously characterized as a pO_2_ reporter molecule for quantitative oximetry using the PISTOL ^1^H MR oximetry technique^38,39^ and HMDSO based nanoemulsions have also been used to report oxygenation *in vivo* and *in vitro* at 4.7 T^44,45^. Furthermore, PDMSO nanoemulsions have been used for cell labelling and oximetry of neural stem/progenitor cells^46^ at 7 T. In this study we had several goals. Firstly, we aimed to expand the utility of the PISTOL technique for MR oximetry by identifying other siloxanes for use as pO_2_ reporter molecules. Temperature-dependent calibration of HMDSO was conducted at 7 T and 9.4 T to study the effect of magnetic field on calibration of HMDSO and to extend its previously demonstrated utility as pO_2_ sensor (at 4.7 T) to higher fields (7 T and 9.4 T). Based on initial observations at 4.7 T, the promising pO_2_ reporter OMTSO was further evaluated at higher fields (7 T and 9.4 T) and calibration curves were characterized as a function of temperature. Finally, given the prior application of PDMSO at 7 T for cell labelling and oximetry^46^, its calibration curve at 7 T was characterized as a function of temperature.

At a given temperature and magnetic field the linear relationship between pO_2_ and temperature (defined by Eq. 3) determines the sensitivity of R_1_ to changes in pO_2_. The intercept A′ represents the relaxation rate observed under anoxic condition (the diamagnetic, oxygen independent contribution R_1D_) and its inverse represents maximum T_1_ displayed by the probe. The slope B′ represents the sensitivity of the probe’s relaxation rate to the changes in oxygenation and is a ratio of the paramagnetic contribution (R_1p_) of oxygen to the relaxation rate of the probe and the solubility of oxygen in the probe. The ratio η = slope/intercept (B′/A′) is a parameter that helps in determining and comparing the sensitivity index of different MRI pO_2_ reporter molecules. A larger slope B′ and smaller intercept A′ represent greater sensitivity to changes in pO_2_ but also indicates longer imaging times. Since a smaller A′ implies a larger maximum T_1_ (observed under anoxic or hypoxic conditions which are usually of interest in studying pathologies), an adequate sampling of the recovery curves would require the use of longer recovery time TR for imaging. We characterized the relaxation behavior of the linear and cyclic siloxanes by comparing the magnetization recovery curves after bubbling with N_2_ (0% O_2_) vs 21% O_2_ (Supplementary Fig. S1). Bi-exponential T_1_ behavior was not observed in any of the magnetization recovery curves of the evaluated linear and cyclic siloxanes suggesting that the availability of oxygen to all the protons (e.g end chain vs backbone for linear siloxanes) was unhindered. We observed a decrease in η with respect to an increase in chain length of the linear siloxanes and the η values ranged from 8.2–11.6 × 10^−3^ torr^−1^ with only small changes in pO_2_ sensitivity. Another important observation was that anoxic relaxation rate (A′) increased with increasing chain length of the siloxanes and was higher for cyclic siloxanes than the linear siloxanes. This is again consistent with the observations for perfluoroalkanes^50^ and alkanes^51^ and is a consequence of the reduction of molecular tumbling rate for larger chain lengths leading to an increase in relaxation rate. The higher anoxic relaxation rate indicates a shorter maximum T_1_ and hence potentially less time needed for T_1_ mapping for the larger siloxanes which can be further exploited to map tissue oxygenation faster than HMDSO. The B′ value remained similar between the linear and cyclic siloxanes indicating that the solubility of oxygen and its proximity to the methyl protons remains similar between the siloxanes of different chain lengths and structures.

Performing MR oximetry at higher magnetic field strengths has the following advantages: 1) resonances have larger chemical shift separation between them which aids in selective excitation of the siloxane resonance as well as the suppression of the water and fat resonances, 2) increase in net magnetization, leading to improved signal-to-noise, 3) improved dynamic range in T_1_ over the physiological pO_2_ range resulting in more accurate pO_2_ measurements. On the other hand, the relaxation times tend to increase at higher magnetic fields^52,53^ which might result in an increase in the total imaging time for pO_2_ mapping. Our goal was to evaluate the relationship of T_1_s of HMDSO and OMTSO at 7 T and 9.4 T and also help in determining the choice of siloxane for applications requiring higher temporal resolution. Our results suggest that at 37 °C for HMDSO, T_1_ was constant at 9 s at pO_2_ = 0 torr and ranged from to 3.5–3.2 s at pO_2_ = 160 torr on changing the field strength from 7 T to 9.4 T, which differed by ~ 2% from T_1_ obtained at 4.7 T^38^. Also, the calculated relative error in pO_2_ determination as given by Eq. [8] at 37 °C was ~ 0.7 torr/°C at 7 T and ~ 1 torr/°C at 9.4 T when the actual pO_2_ value was 5 torr. Since the maximum T_1_ (and hence potentially imaging time) at 7 T and 9.4 T was same as for 4.7 T with no significant increase of temperature-fluctuation induced error in pO_2_ determination, PISTOL oximetry using HMDSO would be improved at higher magnetic field strengths. Similarly, for OMTSO the T_1_ ranged from 6.3–6.8 s at pO_2_ = 0 torr and around 2.8 s at pO_2_ = 160 torr over the range of magnetic fields strengths studied. Thus, the changes in the fields strength will not result in a substantial increase in the imaging time for OMTSO and the η and relative signal (α^siloxane^) are similar to that of HMDSO (Table 1). Further, the calibration of OMTSO and PDMSO demonstrated that longitudinal relaxation rate of both the siloxanes varied linearly with respect to changes in pO_2_ at temperatures in the physiological range, demonstrating the potential of OMTSO and PDMSO to measure dynamic changes in tissue pO_2_. At a temperature of 37 °C OMTSO and PDMSO had an oxygen sensitivity similar to HMDSO (B′ values ranging from 0.0011 to 0.0013 s^−1^ torr^−1^) at all three fields. It should be also noted that boiling point of OMTSO (153 °C) is higher than the boiling point of HMDSO (101 °C).This suggests that it maybe be more advantageous to use OMTSO for generating nanoemulsions for cell labelling applications than HMDSO (used previously^45^) as it would be less volatile during the emulsification process^54^. The simulated errors in pO_2_ determination due to temperature fluctuations for OMTSO as well as PDMSO were found to be in the same range as HMDSO at 4.7 T, 7 T and 9.4 T.

We have previously demonstrated the feasibility of *in vivo* pO_2_ mapping following intra-tissue injection of ‘neat’ HMDSO as well as HMDSO based nanoemulsions^39,44,55^. Dilution of the siloxane in a solvent can potentially affect the pO_2_ calibration curve (and hence T_1_ or R_1_) in a “concentration-dependent” manner by changing the intercept due to changes in the dipole-dipole interactions of the siloxane protons with the solvent protons, although this was not tested here. Jamrogiewicz *et al*. studied the dependence of ^1^H relaxation times of linear HMDSO on dilution using a mixture of carbon tetrachloride with deuterated benzene and found that T_1_ did not significantly depend on the analyte concentration in the sample or the mutual ratio of the solvents used^48^. Dilution of siloxanes in solvents can also affect the slope of the calibration curve as the oxygen solubility may change based on solvent used and the siloxane concentration. Dilution in tissue by use of less siloxane administered per tissue volume or by diluting a siloxane emulsion is unlikely to affect the calibration as the siloxane is restricted to a local partitioned environment consisting solely of other siloxane molecules and dissolved gasses in either case. We recommend using undiluted probes for pO_2_ mapping applications in order to maintain the highest signal to noise ratio.

In general, siloxanes are considered non-toxic or minimally toxic and toxicity decreases with increasing molecular weight^47^. However, individual siloxanes should be evaluated for safety before *in vivo* use. Previous studies have shown that HMDSO is quite inert, and exhibited minimal toxicity in rats tested for subchronic inhalation toxicity^56,57^. No oral toxicity (LD_50_ > 5 ml/kg) was found in rats and no irritation and acute toxicity was reported in Draize tests of skin or eye irritancy in a study in rabbits^58^. In our previous studies, we saw no overt signs of toxicity, inflammation or discomfort after injection of HMDSO^39^ or HMDSO nanoemulsions^44^ into muscle, although no microscopic analyses were performed. Cytotoxicity analysis of HMDSO nanoemulsions showed that the half maximal inhibitory concentration (IC_50_) at a concentration of 0.4–1% (v/v)^45^ in 3T3 fibroblast cells while a IC_50_ > 2% (v/v) was reported for PDMSO nanoemulsions in mouse neural progenitor/stem cells^46^. These findings indicate that the use of siloxanes, especially longer chain siloxanes may be feasible for human applications. In particular, the use of siloxane emulsions for labelling transplanted cells and monitoring cell health is promising due to the trace amounts used. Further, the PISTOL technique used for ^1^H MR oximetry in conjunction with siloxanes utilizes pulse sequence components that are readily available on clinical MRI scanners such as selective RF pulses (for excitation of the siloxane resonance and suppression of fat and water signals) and echoplanar readout (for fast T_1_ mapping). This adds to the promise of clinical translation of ^1^H MR oximetry using siloxanes.

In summary, we have demonstrated for the first time the feasibility of various linear and cyclic siloxanes as pO_2_-sensing probes for ^1^H MR oximetry. Of these OMTSO can be identified as a promising pO_2_ probe which could enable faster mapping of tissue oxygenation than HMDSO without a significant drop in sensitivity. Alternatively, for applications requiring better temporal resolution or for cell labelling applications, one can use cyclic or long chain linear siloxanes, such as PDMSO, along with a recently developed pulse sequence for faster ^1^H MR oximetry^55^. In general, all the siloxanes studied here, with a broad range of boiling points and dynamic range of T_1_’s, can be used for diversifying the applications of ^1^H MR oximetry.

## Materials and Methods

The linear siloxanes HMDSO, OMTSO, DMTSO, DDMPSO, and cyclic siloxanes OMCTSO and DMCPSO were purchased from Sigma-Aldrich (St Louis, MO). PDMSO (MW = 410, viscosity = 2 cSt) was purchased from Alfa Aeser (Tewksbury, MA). All the materials were used as received and all the experiments were conducted without any dilutions i.e. used ‘neat’.

For the sample preparation, each siloxane (1 ml) was placed in 4 gas-tight NMR glass tubes (Wilmad Taperlok, Buena, NJ) and saturated by bubbling for 15 minutes with varying standard of gases including 0%, 5%, 10%, and 21% O_2_ (balance N_2_), respectively. Gases with varying oxygen concentrations were made by mixing nitrogen and air in varying proportions in a HypoxyDial (STARR Life Sciences Corp.; Oakmont, PA). A pO_2_ meter was connected in line with the output of the HypoxyDial in order to verify the accuracy of the HypoxyDial. The tubes were then sealed. For measurement of the temperature dependence of T_1_, the temperature of the water pad was varied between 17 to 52 °C. A fiber optic probe (FISO Technologies Inc., Quebec, Canada) was used to measure the temperature of the tubes.

MR experiments were performed on a Varian Inova 4.7 T, Bruker BioSpec 7 T and Varian Inova 9.4 T. The tubes were placed together on a pad with circulating water and T_1_ measurements were performed using previously described methods^38,39^ after the tube temperature was allowed to equilibrate at the desired value for 10–20 mins using a surface or volume coil. Briefly, T_1_ measurement was conducted by using pulse-burst saturation recovery with a variable TR ranging from 0.1–55 ms. T_1_ data were fit to a single exponential, 3-parameter magnetization recovery equation using the Levenberg-Marquardt algorithm. The data at each temperature was then fit into the Eqs. [4–7] described earlier to obtain the corresponding calibration constants and T_1_ values. MATLAB R2018b (MathWorks, Natick, MA) was used to analyze the images and compute T_1_ maps and pO_2_ maps. Using equation [8], the dependence of errors in pO_2_ determination per 1 °C change due to temperature fluctuations was simulated for oxygenation levels in relevant hypoxic range (0 torr to 50 torr) at 37 °C.

## Supplementary information

Supplementary information.
