# Supplementary material for: More bullets for PISTOL: linear and cyclic siloxane reporter probes for quantitative 1H MR oximetry
Source: Sci Rep. 2020 Jan 29;10:1399. doi: 10.1038/s41598-020-57889-9 (PMC6989524; doi:10.1038/s41598-020-57889-9)
Supplement: Supplementary file 1 — Supplementary information. [file 41598_2020_57889_MOESM1_ESM.pdf]

Supplementary Data

Title: More bullets for PISTOL: linear and cyclic siloxane reporter probes for quantitative ^{1}H MR oximetry

Authors: Shubhangi Agarwal, Praveen K. Gulaka, Ujjawal Rastogi and Vikram D. Kodibagkar

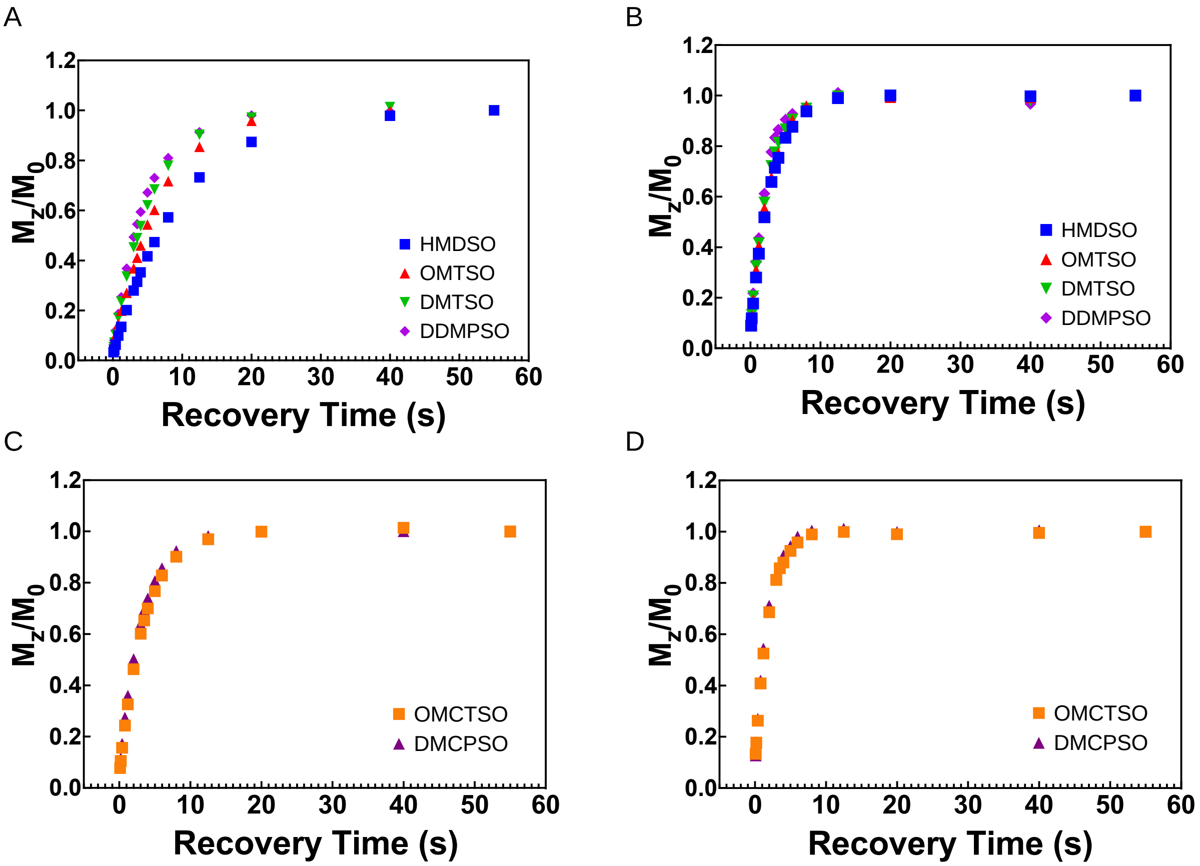

Supplementary figure S1: *Magnetization recovery curves (at 4.7T) of the linear siloxanes after bubbling with* ***(a)*** *0% O_2 and* ***(b)*** *21% O_2 and cyclic siloxanes after bubbling with* ***(c)*** *0% O_2 and* ***(d)*** *21% O_2. These recovery curves do not show a bi-exponential behavior even for linear long chain siloxanes.*

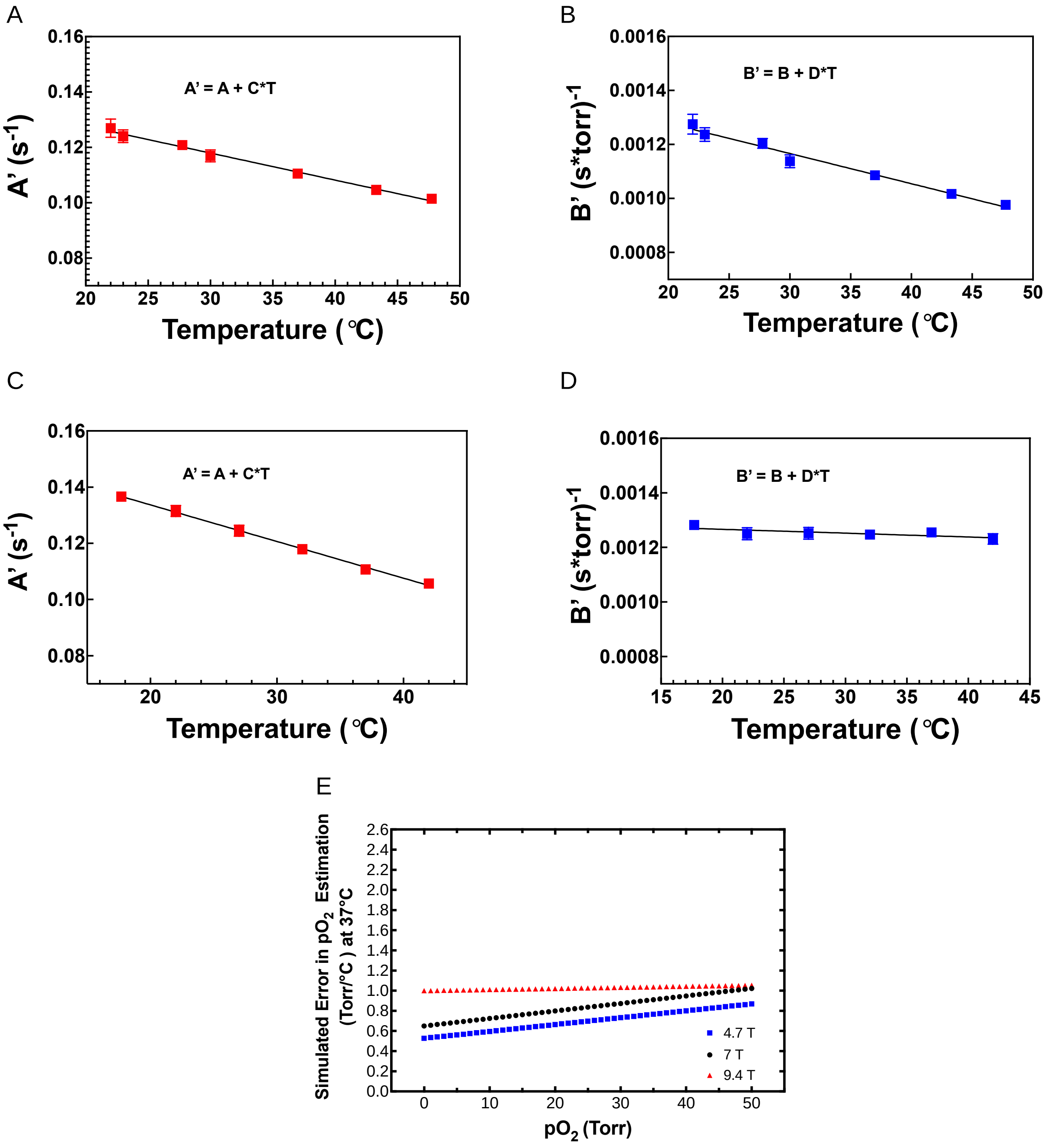

Supplementary figure S2: *Temperature dependence of calibration constants A' and B' of HMDSO at 7 T (**A**, **B**) and 9.4 T (**C**,**D**). (**E**) Comparison of simulated error (equation [8]) in pO_2 determination for HMDSO in the physiological pO_2 range.*

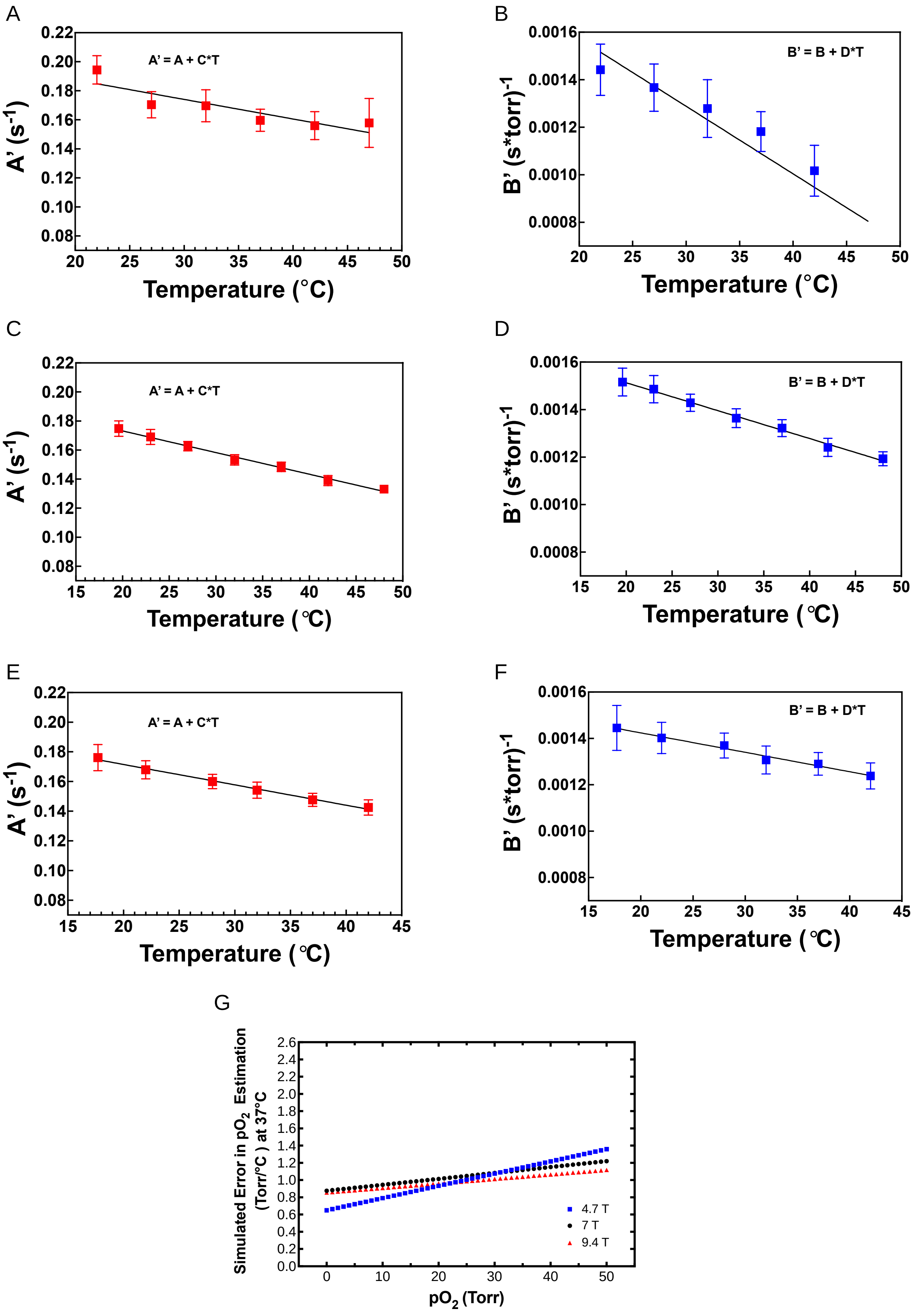

Supplementary figure S3: *Temperature dependence of calibration constants A' and B' of OMTSO at 4.7 T (**A**, **B**), 7 T (**C**,**D**) and 9.4 T (**E**, **F**). **(G)** Comparison of simulated error (equation [8])) in* pO_2 *determination for OMTSO in the physiological* pO_2 *range.*

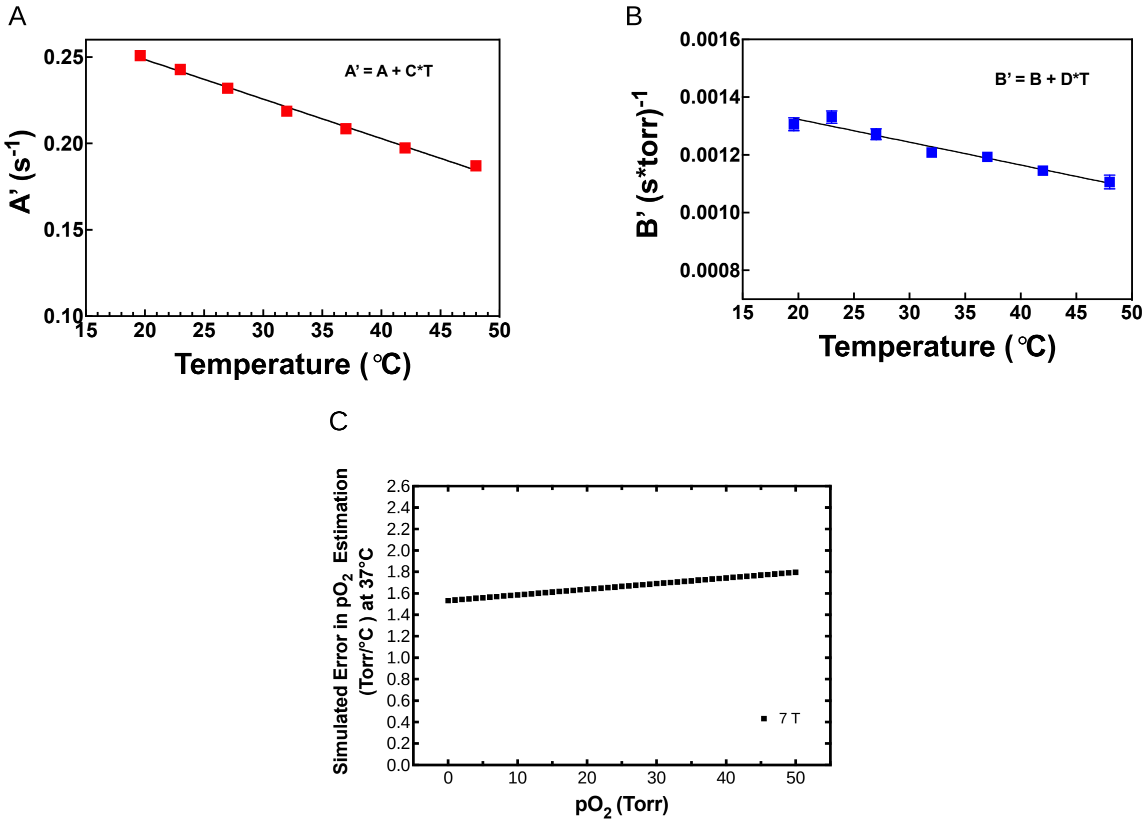

***Supplementary figure S4:** Temperature dependence of calibration constants A' and B' of PDMSO at 7 T (**A**, **B**). **(C)** Simulated error (equation [8])) in pO_2 determination for PDMSO in the physiological pO_2 range.*
